# Comprehensive literature review of oral and intravenous contrast-enhanced PET/CT: a step forward?

**DOI:** 10.3389/fmed.2024.1373260

**Published:** 2024-03-19

**Authors:** Gilles Metrard, Clara Cohen, Matthieu Bailly

**Affiliations:** ^1^Nuclear Medicine Department, Orléans University Hospital, Orléans, France; ^2^Centre de Biophysique Moléculaire, CNRS UPR 4301, Université d’Orléans, Orléans, France; ^3^Radiology Department, Orléans University Hospital, Orléans, France

**Keywords:** PET/CT, iodine, contrast enhanced, one-shot, examination

## Abstract

The integration of diagnostic CT scans into PET/CT facilitates a comprehensive single examination, presenting potential advantages for patients seeking a thorough one-shot check-up. The introduction of iodinated contrast media during PET scanning raises theoretical concerns about potential interference with uptake quantification, due to the modification of tissue density on CT. Nevertheless, this impact appears generally insignificant for clinical use, compared to the intrinsic variability of standardized uptake values. On the other hand, with the growing indications of PET, especially ^18^F-FDG PET, contrast enhancement increases the diagnostic performances of the exam, and provides additional information. This improvement in performance achieved through contrast-enhanced PET/CT must be carefully evaluated considering the associated risks and side-effects stemming from the administration of iodinated contrast media. Within this article, we present a comprehensive literature review of contrast enhanced PET/CT, examining the potential impact of iodinated contrast media on quantification, additional side-effects and the pivotal clinically demonstrated benefits of an all-encompassing examination for patients. In conclusion, the clinical benefits of iodinated contrast media are mainly validated by the large diffusion in PET protocols. Contrary to positive oral contrast, which does not appear to offer any major advantage in patient management, intravenous iodine contrast media provides clinical benefits without significant artifact on images or quantification. However, studies on the benefit–risk balance for patients are still lacking.

## 1 Introduction

A recent editorial has brought forth the question: “PET/contrast-enhanced CT in oncology: to do, or not to do, that is the question” (1).

Positron Emission Tomography (PET) is a molecular imaging technique that enhances diagnostic performance (2), therapeutic response monitoring (3), prognosis evaluation (4, 5) and modifies the management of patients with hematological (6) or solid malignancy (7). However, it is sensitive to attenuation. Unlike Single Photon Emission Tomography (SPECT), PET reconstruction needs the detection of two simultaneous 511 keV photons produced by β + annihilation. The increased interaction probability of at least one of two photons versus only one results in a decreased sensitivity with patient’s depth.

This attenuation has been historically corrected with a 511 keV attenuation map generated using a ^68^Germanium transmission source. The primary drawback of this technique was its inherent slowness, significantly elongating the examination duration. Subsequent PET generation introduced a shift where attenuation correction map was substituted with a simulated high-energy attenuation map derived from Computed Tomography (CT). The first hybrid PET/CT systems offered enhanced accuracy in pinpointing the anatomical uptake location.

Gradually, PET manufacturers integrated the same diagnostic CT used as in radiology. With the potentially comparable performances capabilities, the redundancy between radiological CT and PET/CT prompted physicians to enhance the CT parameters of PET for minimizing overall patient’s exposure, especially in oncology, and the economic impact (8).

The quest to the one-stop-shop anatomic and metabolic exam potentially required contrast-enhanced CT but the attenuation map modification by contrast medium could potentially lead to PET artefacts (9, 10). In this article, we propose a comprehensive literature review on the physical and clinical impacts of intravenous and positive oral contrast-enhanced CT in PET (cePET/CT).

## 2 Physical impact on standardized uptake value

The standardized uptake value (SUV) is the main quantitative parameter in PET to assess the radiopharmaceutical concentrations in tissues, while accounting for radioactive decay.

SUV body weighted (SUV_bw_ or SUW) is determined by the ratio of the activity concentration in the tissue under examination to the activity concentration in the entire body.

However, SUV_bw_ assumed a uniform distribution of the radiopharmaceutical throughout the body which was not the case. Particularly for the mainly used radiopharmaceuticals, the activity level in white adipose tissue was considerably low and led to an SUV_bw_ overestimation in patients with obesity (11). To rectify this, a more accurate approach involved scaling the SUV according to the lean body mass (SUV_lbm_ or SUL) (12) for adults or to the body surface area (SUA) in pediatric patients (13).

Furthermore, various other factors could potentially interfere with radiopharmaceutical distribution, such as injected activity, post-injection uptake time, blood glucose level, attenuation correction (14).

There was also multiple methods for quantifying SUV in a region of interest (ROI). SUV_mean_ represented the average activity in the ROI while SUV_max_ captured only the maximum pixel value. However, SUV_max_ was more sensitive to noise (15) and SUV_mean_ tended to lower the quantitative values with a better repeatability (16). An alternative approach could involve SUV_peak_ which computed the mean pixel value in the vicinity of the pixel with the maximum value and is less sensitive to changes in reconstruction parameters (17).

### 2.1 Intravenous iodine contrast media

On anthropomorphic phantom with and without Iodine Contrast Media (ICM), the study conducted by Razac et al. revealed a marginal absolute difference ΔSUV_max_ and ΔSUV_mean_, of 0.2 and 0.4, respectively (18). This disparity was more pronounced in the most metabolically active simulated lesion (ΔSUV = 1.5 for a SUV_max_ of 22). Nevertheless, these discrepancies were not clinically or statistically significant. These findings corroborated those of Bunyaviroch et al. which indicated a maximum SUV relative difference of 7% on phantom studies and a lower variance with 5.9% in clinical application (19). A variability of up to 8% was also found in conventional and digital systems complying with EARL accreditation but a more than 30% SUV difference could be observed on a limited number of lesions (17).

In clinical practice, the SUV fluctuation was heightened in highly contrast-enhanced regions, although these differences were not deemed significant (20, 21). Even when the variability was statistically significant, the authors did not observe any discernible clinical impact (22). This ICM SUV induced difference remained relatively negligible compared to the 20–30% global SUV variability in tumors (16). For this reason, it was recommended to use a 25% SUV decreased threshold for tumor reduction and a 33% SUV increased threshold for tumor progression in follow-up (23).

To mitigate the effects of ICM on attenuation correction, numerous research groups have explored how to refine the injection protocol, such as adjusting the ICM dose, concentration, or flow rate.

Regarding dose adjustment, an adaptation to the body surface area demonstrated a decrease in SUV variability, compared to a fixed-dose approach, and an improved interpatient homogeneity of contrast enhancement (24).

Similarly, the same researchers compared the effects of ICM dosage, finding no significant difference between 300 and 370 mgI/ml (25). When using an even higher iodine concentration of 400 mgI/ml, in a multiphase contrast enhanced CT protocol, Aschoff et al. noted only a minimal to negligible influence on 2-[^18^F]FDG (^18^F-FDG) quantification (26). It was advisable to opt for a single-phase CT rather than a multiphasic protocol to minimize coregistration errors (27).

### 2.2 Oral contrast media

Similarly to ICM, positive oral contrast agents could influence SUV values. This effect was demonstrated on phantom with SUV overestimation for high-density oral contrast agent (28) and the absence of significant artifact for low-density barium oral contrast agents (29). However, in a small patient cohort, Otsuka et al. did not find a significant correlation between SUV and Hounsfield density (30).

On simulated PET reconstructions, Dizendorf et al. demonstrated that oral contrast agents overestimated PET attenuation coefficients by 26.2% with only a small effect on SUV PET. The error was measured at 4.4% and did not appear to be clinically significant (31).

## 3 Additional risks of contrast media in cePET/CT

In clinical use and because of their route of administration, positive oral contrast media were remarkably safe and side effects were exceedingly rare (32). Most complications were observed with intravenous contrast agents.

ICM allowed the enhancement of vascular structures and tissue contrast. They were classified into two major categories: highly hyperosmolar ICM and hypo- or iso-osmolar non-ionic ICM. Both types could induce side effects, with a higher prevalence seen in ionic ICM. This discrepancy in side effects prevalence was the reason why non-ionic ICM were preferred (33).

Warming iodine contrast media at 37°C could also reduce the risk of allergic or physiologic reactions (34).

### 3.1 Pseudo-allergic and allergic reactions

While ionic ICM previously resulted in side effects for 12% of patients, the use of non-ionic ICM had significantly decreased this occurrence to 0.7–3.1% and the most severe reactions have been drastically reduced from 0.22 to 0.02–0.04%.

ICM reactions were mostly non-fatal and manifested in 70% of patients within the first 5 min following ICM injection (35).

The majority of ICM reactions were non-allergic. Hyperosmolality induced fast vascular volume changes or direct chemotoxicity which could lead to physiological responses such as flushing, nausea and altered taste. Another reaction came from a non-allergic hypersensitivity caused by the direct release of histamine from mast cells and basophils. This mechanism could result in allergic-like symptoms like urticaria. For mild reactions, simple monitoring or H1-antihistamines treatment was generally sufficient.

IgE-mediated allergy was uncommon. In more severe cases involving laryngeal edema, corticosteroid therapy was often initiated while resuscitation measures were implemented during anaphylactic shocks (36).

For at-risk patients, a prophylactic treatment could be implemented (H1-antihistamines, corticosteroids) (37).

### 3.2 Nephrotoxicity

ICM increased the risk of acute kidney injury within 48 h following injection. This risk, previously overestimated, could now be prevented by hydration when clearance was below 30 mL/min/1.73 m^2^ or for high-risk patients without contra-indication (38). As contrast enhanced CT was optional in PET, it might be advisable to refrain from administering ICM to these patients. For the specific case of myeloma, a meta-analysis suggested that no special precaution was needed if the calcium level was within the normal range (39).

### 3.3 Metformin

Metformin is an oral antihyperglycemic medication commonly prescribed for diabetes. In the context of ICM injection, patients might potentially develop lactic acidosis coupled with renal failure (35). The European Society of Urogenital Radiology (ESUR) guidelines and American College of Radiology manual recommended discontinuing the treatment for 48 h and monitoring renal function when the baseline clearance was below 30 mL/min/1.73 m^2^ or if there were signs of acute renal failure.

### 3.4 Extravasation

Compared to the low injected volume of radiopharmaceuticals, ICM injection is carried out at higher pressure and for a larger volume. The risk of extravasation reported in the literature ranged from 0.1 to 0.9% and was increased when using an automatic injector (40, 41) or in cancer patients (42).

The risks associated with extravasation increased with osmolality but also depended on its direct toxicity. This toxicity was notably more pronounced with ionic ICM, as well as the anatomical location or volume. While most cases were relatively benign resulting in minor issues like skin erythema, there was potential for more severe side effects such as compartment syndrome or necrosis (40).

The widespread use of non-ionic ICM usually did not expose patients to the risk of severe complications. Therefore, a surgical consultation might be advised only for volumes exceeding 150 mL or in case of compressive signs (impaired perfusion or altered sensibility) (40).

### 3.5 Contrast-induced thyroid dysfunction

A typical radiological dose of ICM contains a substantial amount of free iodine, equivalent to the iodine needs for several months. When the body encountered excess iodine, the Wolff-Chaikoff effect was triggered, causing a fast downregulation in thyroid hormone synthesis. Prolonged exposure to high iodine levels could disrupt this regulatory mechanism, potentially resulting in either hyperthyroidism or hypothyroidism.

Moreover, this excess iodine load had the potential to exacerbate existing thyroid pathology or even directly cause thyroid toxicity (43).

### 3.6 ICM transformation products and potential toxicity

While ICM themselves were not inherently toxic, their presence in source waters raised concerns due to the formation of potentially toxic transformation products detected in drinking water (44). Specifically, ICM could react with commonly used disinfectants like chlorine, leading to formation of iodinated disinfection byproducts (iodo-DBP). Studies indicated that these iodo-DBP were highly genotoxic or cytotoxic, surpassing the conventional DBPs in toxicity. This situation highlighted concerns regarding the effectiveness of current treatment technologies and raised serious questions about disinfecting water containing ICM (45). Recent proposals suggested measures aimed at reducing and collecting ICM residues (46).

These potential side effects needed to be balanced against the clinical benefits of an enhanced CT for the patients. Table 1 summarizes the main advantages and disadvantages of ICM injection in PET/CT.

**Table 1:** Main advantages and disadvantages of cePET/CT.

Pros	Cons
Better lesion contrast especially in low-contrast lesions Better delineation of anatomic structures (digestive and vascular structures, liver, muscles…) Better PET/CT performance: sensitivity, specificity, accuracy Better visualization of urinary tract More comfortable for the patient Overall cost lower than 2 exams	A more complex exam scheduling: kidney function, allergies, medications Additional risks: pseudo-allergic and allergic reactions, kidney failure… Artefacts with SUV overestimation No additional reimbursement in many countries

## 4 Clinical added value of cePET/CT

### 4.1 Head and neck tumors

Squamous cell carcinoma (SCC) represents the most common head and neck tumor type (95%). ^18^F-FDG cePET/CT with dual phase has been proved superior to conventional imaging by MRI or CT for diagnosis and staging of patients with laryngeal carcinoma, with an higher rate of regional nodal, distant metastasis, and synchronous tumors (5, 47). More globally, ^18^F-FDG PET/CT is a recognized modality for the staging and follow-up of head and neck SCC (48, 49). In cases with cervical lymph node metastasis from an unknown primary tumor, ^18^F-FDG PET/CT revealed primary tumors that went undetected by CT or MRI in about 25% of cases (50). However, in those studies the difference between PET/CT and cePET/CT was not evaluated.

Prognosis for head and neck SCC is partly influenced by Human Papilloma Virus (HPV) status, with evidence that virally induced tumors responded far better to radiotherapy (51, 52). Using the hypoxia-specific tracer ^18^F-fluoroazomycin arabinoside (FAZA), Saksø et al. demonstrated that the risk of locoregional recurrence was higher among patients with more hypoxic, non-HPV tumors (57% [21–94%]), when comparing to less hypoxic, non-HPV tumors, with a risk difference of 45% [4–86%] (53).

Integrated ^18^F-FDG PET/perfusion CT showed that tumoral perfusion was significantly increased compared to surrounding soft tissue, especially for advanced tumors, and that meant blood flow was decreased in HPV-negative tumors (54).

Suenaga et al. showed that cePET/CT and PET/CT statistically showed larger AUC than contrast enhanced CT (ceCT) for recurrent head and neck squamous cell carcinoma (55). Even though minimal, the difference between cePET/CT and PET/CT for local recurrence reached a significant level (*p* = 0.039).

These works highlighted the distinctions between HPV-positive and negative tumors and emphasized the utility of analyzing the microvasculature features of tumoral head and neck SCC to predict their aggressiveness. This illustrated the necessity of integrating non-morphologic parameters, and also looking beyond the SUV uptake.

### 4.2 Digestive tumors

^18^F-FDG ceCT had a higher predictive positive value for any PET pathologic findings than CT in the whole gastrointestinal tract, as well as in the separate evaluation of the upper and lower gastrointestinal tract (56). The sensitivity for the detection of a malignant lesion was 100% for ceCT and 29.4% for CT (*p* = 0.0001). The false negative rate for any pathology was 31.1% for ceCT and 68.9% for CT; this rate was however lower in the lower gastro-intestinal tract for CT (12.5% vs. 37.5% for ceCT).

^18^F-FDG PET and ceCT seemed to have similar value in the detection of unsuspected recurrence of high-risk colorectal cancer in a patient-based analysis: sensitivity and specificity of 86 and 88%, 86 and 92%, 86 and 85%, respectively for PET, ceCT and cePET/CT (57). However, the combined assessment of cePET/CT improved the accuracy in the lesion-based analysis: sensitivity of 56, 71 and 97%, respectively for PET, ceCT and cePET/CT.

Regarding rectal tumors, cePET/CT was superior to non-enhanced PET/CT for precise definition of regional nodal status in rectal cancer with a better characterization of pararectal, internal iliac and obturator lymph nodes (58).

A retrospective study explored the diagnostic value of the cross-modality fusion images provided by ^18^F-FDG PET/CT and ceCT in differentiating malignant from benign pancreatic lesions and staging pancreatic cancer (59). The authors found higher sensitivity and accuracy of cePET/CT compared to PET/CT and ceCT conducted individually both for diagnosing pancreatic malignant tumor and peripancreatic vessel invasion. Regarding regional lymph node metastasis, there was no significant differences between the three methods: however, regarding distant metastasis, cePET/CT improved sensitivity and negative predictive value in comparison to ceCT alone. cePET/CT had also higher sensitivity and accuracy than PET/CT, but the difference was not statistically significant.

Considering neuroendocrine tumors, a recent study emphasized that ceCT in ^68^Ga-DOTATATE PET should be included for staging. The overall lesion-based sensitivity, specificity, negative predictive value and positive predictive value were 97% versus 85, 86% versus 73, 93% versus 72, 93% versus 85%, respectively, for full-dose cePET/CT and low dose PET/CT (60).

In the case of positive oral contrast media, several studies have demonstrated an improvement in digestive distension which was a potential help for diagnosis (61–63), but few have been able to demonstrate a clinical benefit. Chen et al. reported a more accurate delayed PET/CT with laxative-augmented contrast medium than conventional PET/CT for the evaluation of colorectal foci (64) and Guo et al. reported a case of enterovesical fistula revealed with oral contrast (65). The main challenge in assessing the impact of oral contrast agents was the hyperfixation of digestive structures (66), particularly due to distension, increased motility, and irritative phenomena (30). Regarding these digestive fixations, they were less pronounced with negative oral contrast agents (67–69) or those with low iodine density (62, 70).

### 4.3 Gynecological tumors

Considering malignant ovarian tumors, ^18^F-FDG cePET/CT outperformed ceCT with sensitivity, specificity, negative predictive value, positive predictive value, and accuracy of 96 versus 84%, 92 versus 59%, 90 versus 59%, 97 versus 84%, and 95 versus 76%, respectively for cePET/CT and PET/CT alone (71). cePET/CT represented an accurate imaging modality for staging ovarian cancer (72).

Regarding ovarian cancer recurrence, cePET/CT seemed to be more accurate. Some data suggested higher sensitivity, specificity, and accuracy of cePET/CT: 86.9, 95.9, and 92.5%, respectively, (compared to 78.3, 95.0, and 88.3%, respectively for PET/CT) (*p* = 0.023 at least) (73). Another study found a better identification of smaller peritoneal/lymph node lesions close to physiological FDG uptake sources with cePET/CT (74). With a better accuracy compared to non-contrast PET/CT and enhanced CT, cePET/CT could lead to changes in patient management for 39% of them (75).

Similarly, for uterine cancer, cePET/CT seemed to perform slightly better (sensitivity and accuracy) for nodal staging (*p* = 0.046 and 0.047) (76). cePET/CT was accurate for recurrence, reducing the frequency of equivocal interpretations (77) and leading to more appropriate subsequent clinical management than that resulting from PET alone or ceCT alone (78).

### 4.4 Melanoma

A previous study recommended to perform ^18^F-FDG PET/CT instead of cePET/CT for staging of malignant melanoma patients (79). Comparison between CT and ceCT alone clearly revealed higher sensitivity and specificity for ceCT. However, when directly comparing lesion-based evaluation of combined PET/CT and cePET/CT, there was a difference in sensitivity of 3% only and no difference in specificity. As a limit, this study was conducted on a non-time-of-flight PET/CT system.

### 4.5 Hematological cancers

Integrated cePET/CT could improve the evaluation of pelvic lymphatic pathways nodal status in patients with malignant lymphoma (external and internal iliac, common iliac lymph nodes); diagnostic accuracies of retroperitoneal lymph nodes seemed to be similar between PET/CT and cePET/CT (80). However, the contribution of ceCT in nodal staging (Ann Arbor) seemed to remain limited (81). Similarly, the response evaluation applying the Deauville score and Lugano classification criteria remained unchanged with cePET/CT (82).

Thus, cePET/CT could be performed in the management of lymphoma patients, especially for a precise target delineation before radiotherapy (83).

CePET/CT approach should also be considered in pediatric exams. It could offer dose savings at similar image quality for children and young adults with lymphoma who had indications for both PET and diagnostic CT examinations (84).

### 4.6 Other malignancies

^18^F-FDG cePET/CT showed similar results compared with CT/MRI in the detection of primary renal tumors, but it was superior to conventional methods in the detection of metastasis and staging (85). Once again, in this study, the authors did not compare directly PET/CT and cePET/CT.

Similarly, the same authors also showed higher diagnostic accuracy of ^18^F-FDG cePET/CT for staging bladder cancer (89% vs. 57% for conventional imaging: CT and MRI), with upstaging in 37% of patients, resulting in changes of patient management.

The use of contrast has also been described as useful in ^18^F-FDG cePET/CT as an initial imaging modality in patients presenting with metastatic malignancy of undefined primary origin (86).

In the specific case of lung and breast cancers, although these cancers were frequent, the clinical contribution of iodinated contrast injection has not been studied. It had only been demonstrated for lung cancers that non-ionic contrast injection did not cause significant artifact (21).

**Figure 1:**
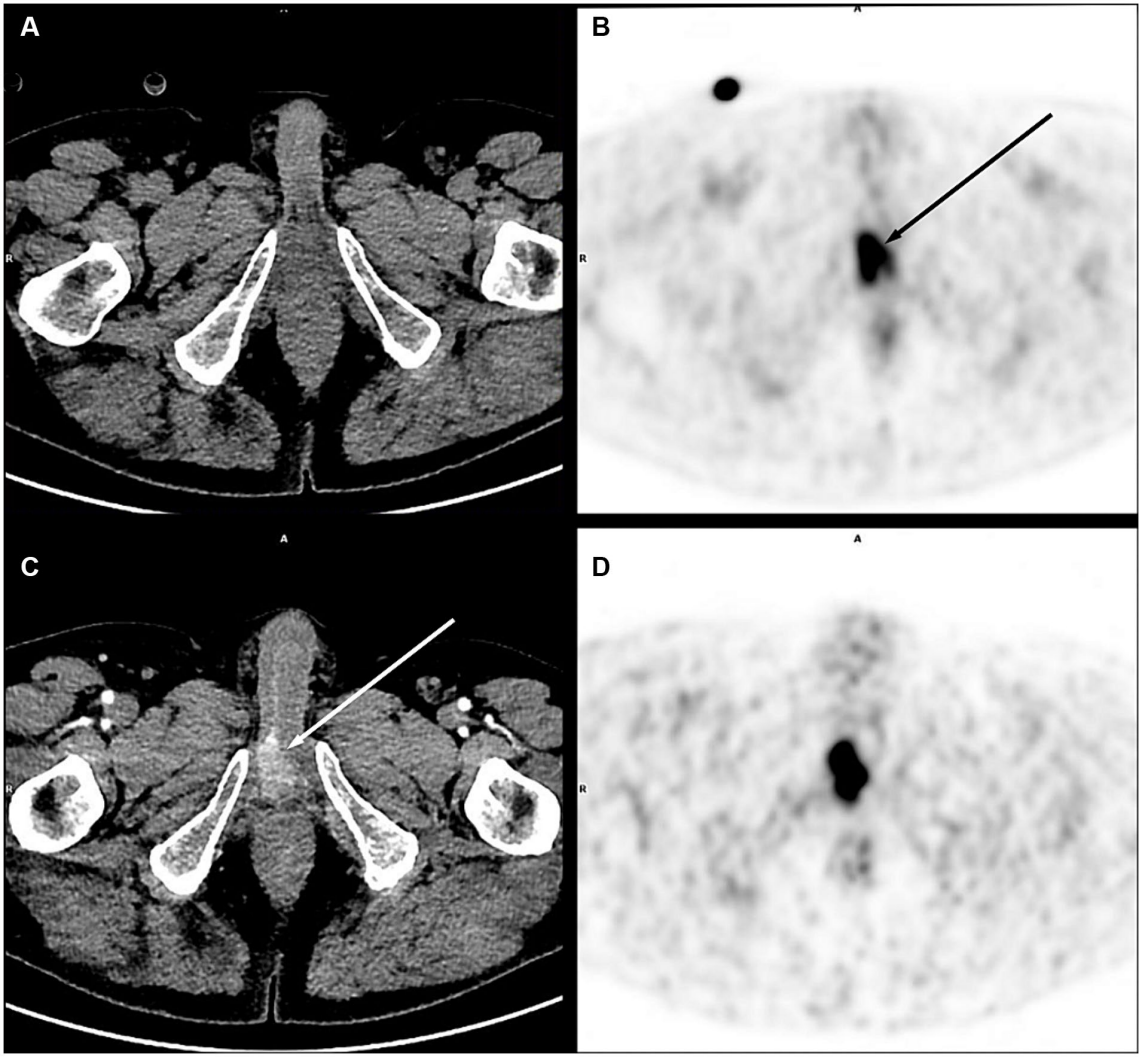
Right corpora cavernosa metastasis from penile carcinoma. on PET images (early acquisition **B**, late acquisition **D**), there was a focal uptake [**(B)** black arrow], without significant lesion on unenhanced CT **(A)**, in an area close to the physiological urinary activity. With contrast medium injection on a dedicated acquisition **(C)**, this uptake corresponded to a metastatic lesion clearly visible on CT (white arrow).

Figure 1 illustrates the improved visualization of low-contrast lesions, especially in difficult areas for diagnosis.

### 4.7 Non-tumor pathologies: inflammation and infection

A recent study focused on the diagnostic challenge in suspected infected aortic aneurysms, showing the high diagnostic accuracy of PET/CT for the detection of infection (sensitivity between 85 and 100% vs. between 63 and 88% for ceCT) (87). However, the authors raised the question of specificity because of false positive findings. The combined acquisition and analysis of PET and ceCT could help to improve this specificity.

In vasculitis, cePET/CT could be useful for identifying stenotic lesions in Takayasu arteritis, but data are insufficient to support its routine use for giant cell arteritis large vessel vasculitis. Guidelines recommended a low-dose CT prior to ceCT for attenuation correction and subsequent SUV calculations (88).

Recent guidelines in the management of infectious endocarditis recommended cePET/CT, as it allowed the detection of metabolic findings (FDG uptake distribution and intensity) and anatomical findings (endocarditis-related lesions like abscess) within a single imaging procedure, resulting in the clinical clarification of indeterminate findings and change in the management of the patients. This might be particularly helpful in complex settings like aortic grafts (89).

Lastly, contrast enhancement with an ICM enabled the detection of others pathologies, mostly not visible in PET/CT, such as lesions below the system resolution or pulmonary embolism, which were common in oncology (90).

## 5 Clinical added value of cePET/CT with other radiotracers

### 5.1 ^18^F-choline

Despite being widely used for prostate cancer in many countries, only a few studies on parathyroid glands have been published. In a meta-analysis, Piccardo et al. found a better pooled sensitivity for 4D cePET/CT compared to PET/CT in primary hyperparathyroidism (Sensitivity of 0.93 and 0.89, respectively) with an identical detection rate (0.86) (91). In a cohort comprising 44 primary hyperparathyroidism patients, the same researchers confirmed the higher sensitivity of cePET/CT over PET/CT (with sensitivity of 1.0 and 0.8, respectively) and a better detection rate of 0.72 compared to 0.56, respectively (92).

### 5.2 ^68^Ga-DOTATOC

For neuroendocrine tumors, ^68^Ga-DOTATOC cePET/CT demonstrated a minimal increase in sensitivity and specificity compared to unenhanced exam (93). Ruf et al. recommended the same multiphase protocol for cePET/CT as for CT scan (94, 95).

### 5.3 ^68^Ga-PSMA ligand

Although the injection of contrast media did not yield a significant difference in diagnostic performance between PET/CT and cePET/CT, contrast enhancement seemed to improve the delineation of genitourinary system and increased the diagnostic certainty and interobserver agreement (96, 97).

However, CT acquisition during the contrast urinary excretion allowed for a better identification of the urinary tract. In a large retrospective study of 247 patients, Rosar et al. demonstrated that CT urography increased diagnostic confidence (in 48.6% of patients) while providing substantial support for interpretation (24.3%). In 12.1% of patients, urography changed the disease staging with a potential impact on patient management (98).

Tulipan et al. also showed that iodinated contrast agent sedimentation in the bladder created an activity gradient that improved visualization of the prostatic bed and the posterior bladder (99).

## 6 Issues raised by the all-in-one PET/CT exam

The one-stop-shop PET exam with ICM blurs the boundary between nuclear medicine and radiology. Depending on the country, this may rise issues of legislation and reimbursement. In addition, the choice of cePET/CT injection protocol is not standardized and differs from one nuclear medicine department to another (100).

## 7 Conclusion

The use of iodinated contrast media (ICM) in PET/CT scans enhanced the overall examination performance by combining the PET sensitivity and specificity with those of diagnostic enhanced CT. This synergistic performance enhancement was achievable through an all-in-one examination, improving patient dosimetry, facilitating pathology management, and decreasing the administered volumes of ICM especially in the field of oncology. In contrast, although positive oral contrast media enhanced distension and contrast of digestive structures, their clinical utility in PET imaging appeared more modest.

However, the ICM injection was not exempt from side effects, most of which were moderate. For the most severe forms, the additional risk remained low, as most patients would have undergone an ICM enhanced CT as part of their assessment. Apart from contraindications, injecting less than 150 mL of non-ionic ICM into patients with a renal clearance greater than 30 mL/min/1.75 m^2^ could maximize the safety of ICM use in PET as long as no benefit–risk studies have been carried out.

## Author contributions

GM: Writing – original draft, Writing – review & editing. CC: Writing – original draft, Writing – review & editing. MB: Writing – original draft, Writing – review & editing.
